# 475. Does Metabolite Matter? Defining Target Itraconazole and Hydroxy-itraconazole Serum Levels for Blastomycosis

**DOI:** 10.1093/ofid/ofac492.533

**Published:** 2022-12-15

**Authors:** Danielle Hess, Omar M Abu Saleh, Mark Enzler, Paul J Jannetto, Kristin Mara, Ryan W Stevens, Paschalis Vergidis, Christina G Rivera

**Affiliations:** Mayo Clinic, Rochester, Minnesota; Division of Public Health, Infectious Diseases, and Occupational Medicine, Department of Medicine, Mayo Clinic, Rochester, Minnesota; Mayo Clinic College of Medicine, Rochester MN, Rochester, Minnesota; Mayo Clinic, Rochester, Minnesota; Mayo Clinic, Rochester, Minnesota; Mayo Clinic, Rochester, Minnesota; Mayo Clinic, Rochester, Minnesota; Mayo Clinic, Rochester, Minnesota

## Abstract

**Background:**

Itraconazole is first-line treatment for mild-moderate blastomycosis and consolidation of moderate-severe disease. Itraconazole is metabolized to 3 metabolites, including an active metabolite hydroxy-itraconazole. The sum of itraconazole and hydroxy-itraconazole levels > 1.0 mcg/mL is guideline recommended for treatment of invasive fungal infections; conversely, some experts suggest targeting a parent compound level alone > 1.0 mcg/mL. This study aims to compare clinical outcomes and adverse drug reactions (ADRs) of combined itraconazole and hydroxy-itraconazole levels > 1.0 mcg/mL versus itraconazole parent compound alone > 1.0 mcg/mL in patients with blastomycosis.

**Methods:**

This study is a single-center, retrospective chart review of patients ≥ 18 years with probable or proven *Blastomyces* infection who received itraconazole with at least one documented serum itraconazole level. The primary outcome was rate of partial or complete treatment response in patients with a combined itraconazole and hydroxy-itraconazole level > 1.0 mcg/mL versus itraconazole parent compound > 1.0 mcg/mL for > 75% of measured levels. ADRs attributable to itraconazole were compared between the groups. Treatment response rates and ADRs were compared between groups using two proportion z-tests.

**Results:**

Total of 80 patients were included: 36 = combined itraconazole and hydroxy-itraconazole > 1.0, 32 = itraconazole parent alone > 1.0, 12 = 75% of all levels < 1.0. No statistically significant difference was observed between groups for blastomycosis rate of partial or complete treatment response (94.3% combined vs 96.6% parent, p=0.99). Significantly higher mortality was observed in patients failing to achieve itraconazole or itraconazole/hydroxy-itraconazole > 1.0 (2.8% combined vs 0% parent vs 25% neither, p=0.01). There was no significant difference in total ADRs between the three groups (p=0.56).

**Conclusion:**

This limited evidence supports an itraconazole therapeutic target combining itraconazole and hydroxy-itraconazole > 1.0 for blastomycosis treatment.

**Disclosures:**

**Paschalis Vergidis, MD**, AbbVie: DSMB|Cidara: Grant/Research Support|Scynexis: Grant/Research Support **Christina G. Rivera, PharmD**, Gilead: Grant/Research Support|Gilead: Honoraria|Insmed: Honoraria.

